# Blood loss due to diagnostic testing in extremely preterm infants in 22 European countries: a prospective observational study

**DOI:** 10.1016/j.eclinm.2026.104035

**Published:** 2026-06-26

**Authors:** Nina A.M. Houben, Suzanne Fustolo-Gunnink, Karin Fijnvandraat, Camila Caram-Deelder, Marta Aguar Carrascosa, Alain Beuchée, Kristin Brække, Francesco Cardona, Anne Debeer, Sara Domingues, Stefano Ghirardello, Ruza Grizelj, Emina Hadžimuratović, Christian Heiring, Jana Lozar Krivec, Jan Malý, Katarina Matasova, Carmel Maria Moore, Tobias Muehlbacher, Miklos Szabó, Tomasz Szczapa, Gabriela Zaharie, Justine de Jager, Nora Johanna Reibel-Georgi, Helen V. New, Simon J. Stanworth, Emöke Deschmann, Charles C. Roehr, Christof Dame, Saskia le Cessie, Johanna van der Bom, Enrico Lopriore, INSPIRE Study Group, Miguel Alsina-Casanova, Ola Andersson, Rosa Patricia Arias-Llorente, Adeline Berenger, Edyta Bielska, Marioara Boia, André Birkenmaier, Jakub Biros, Anne Laure Blanquart, Tiziana Boggini, Pascal Boileau, Renata Bokiniec, Ilia Bresesti, Katherine Broad, Giacomo Cavallaro, Jennifer Chauvel, Borbála Cseszneki, Carlo Dani, Klaudia Demová, Diana Dornis, Marie-Pierre Duban, Karolina Dziadkowiec-Motyl, Nika Erzen, Eszter Fanczal, Sara Fernández-Castiñeira, Libusa Galuschka, Ellen Gandaputra, Fermín García-Muñoz Rodrigo, Corinna Gebauer, Hélène Grimault, Kristina Grund, Melanie Gsöllpointner, Silvia Gualdi, Brunetta Guaragni, Markus Hahn, Nadja Haiden, Monica Hasmasanu, Daniela Iacob, Mihaela Ivanici, Raphaela Jernej, Tomáš Juren, Karolina Karcz, Lilijana Kornhauser, Barbara Królak-Olejnik, Lena Legnevall, Verena Lehnerer, Emmanuelle Levine, David Ley, María Del Carmen López Castillo, Mariella Magarotto, Silvia Martini, Iwona Maruniak-Chudek, Rita Moita, Anjola Mosuro, Agnieszka Nowicka, Daniel O'Reilly, Manuela Pantea, Alejandro Pérez-Muñuzuri, Tina Perme, Laura Picciau, Simone Pratesi, Sandra Prins, Maurizio Radicioni, Genny Raffaeli, Reyes Roldan-López, Jean-Michel Roué, Beata Rzepecka Węglarz, Greta Sibrecht, Pauline Snijder, Mirta Starčević, Emese Szántó, Liliana Teixeira, Laura Torrejon, Lourdes Urquía Martí, Laurien Vanbuggenhout, Lorenzo Zanetto

**Affiliations:** a Sanquin Research, Sanquin Blood Supply Foundation, Amsterdam, the Netherlands; b Leiden University Medical Center, Leiden, the Netherlands; c Amsterdam UMC, University of Amsterdam, Emma Children’s Hospital, Pediatric Hematology, Meibergdreef 9, Amsterdam, Netherlands; d Sanquin Research, Department of Molecular Cellular Hemostasis, Amsterdam, the Netherlands; e La Fe University Hospital, Valencia, Spain; f CHU de Rennes, Rennes, France; g Oslo University Hospital, Oslo, Norway; h Medical University Vienna, Vienna, Austria; i UZ Leuven, Leuven, Belgium; j Centro Materno-Infantil do Norte - Unidade Local de Saúde de Santo António, Porto, Portugal; k Fondazione IRCCS Policlinico San Matteo, Pavia, Italy; l University Hospital Centre Zagreb, University of Zagreb, School of Medicine, Zagreb, Croatia; m University Medical Centre Sarajevo, Sarajevo, Bosnia and Herzegovina; n Department of Neonatal and Paediatric Intensive Care, Copenhagen University Hospital, Rigshospitalet, Copenhagen, Denmark; o University of Ljubljana, Faculty of Medicine, Ljubljana, Slovenia; p University Medical Centre Ljubljana, Ljubljana, Slovenia; q University Hospital Hradec Králové, Hradec Králové, Czech Republic; r Jessenius Faculty of Medicine, University Hospital Martin, Martin, Slovakia; s University College Dublin, Dublin, Ireland; t National Maternity Hospital, Dublin, Ireland; u University Hospital Zurich, Zurich, Switzerland; v Department of Neonatology Semmelweis University, Budapest, Hungary; w II Department of Neonatology, Poznan University of Medical Sciences, Poznan, Poland; x University of Medicine and Pharmacy Iuliu Hatieganu, Cluj-Napoca, Romania; y Charité - Universitätsmedizin Berlin, Berlin, Germany; z NHS Blood and Transplant, London, United Kingdom; aa University of Oxford, Oxford, United Kingdom; ab Karolinska Institute, Stockholm, Sweden; ac National Perinatal Epidemiology Unit, Oxford Population Health, University of Oxford, Oxford, United Kingdom; ad Faculty of Health and Life Sciences, University of Bristol, Bristol, United Kingdom; ae Women’s and Children’s Division, Southmead Hospital, North Bristol NHS Trust, Bristol, United Kingdom

**Keywords:** Blood loss, Preterm infants, NICU, Diagnostic testing, Europe

## Abstract

**Background:**

Extremely preterm infants often develop anemia of prematurity, partly caused by blood losses for laboratory diagnostic tests during their stay in the neonatal intensive care unit (NICU). However, international quantitative data on diagnostic blood loss in extremely preterm infants are limited.

**Methods:**

We performed an international, prospective, observational study across 64 NICUs across 22 European countries (ISRCTN17267090) to describe diagnostic blood losses during the first 28 days after birth in extremely preterm infants born below 28 weeks. Data collected between September 1st, 2022 and August 31st, 2023 (6-weeks per center).

**Findings:**

We included 320 extremely preterm infants (46% female; median gestational age at birth 26 + 1 weeks; median birthweight 800 g). Median estimated cumulative diagnostic blood loss at day 28 in infants born at 24, 25, 26, 27 weeks’ gestation was 49.6%, 25.9%, 19.7%, 11.5% of calculated initial blood volume (assuming 70 mL/kg birthweight), respectively. Median number of laboratory tests ranged from 6.5 to 25 per center after birth (postnatal day 1–2), and median associated diagnostic blood loss on day 1 and 2 combined ranged from 1.6 to 26.7 mL/kg. There was considerable variation between centers in minimum blood volumes required for laboratory testing. Infants admitted to centers with small-volume analyzers experienced half the cumulative diagnostic blood loss by day 28 (8.2 mL/kg), compared to those admitted to centers with medium- and large-volume analyzers (17.3 and 19.9 mL/kg, respectively).

**Interpretation:**

In this cohort study of extremely preterm infants, we found significant diagnostic blood losses, particularly in the first week, resulting in an estimated cumulative loss of half of the initial blood volume in infants born at 24 weeks. Our findings highlight considerable variations between European centers, underlining the need to understand these differences and minimize diagnostic blood losses whenever possible in this vulnerable patient population.

**Funding:**

Sanquin, ESPR, EBA.

**Research in context**

**Evidence before this study**

We searched PubMed for studies assessing blood loss due to diagnostic testing in preterm infants published from January 1st, 2000, to April 1st, 2026, without language restrictions. The search strategy combined the terms “preterm infant”, “blood loss”, and a term related to the source of blood loss (“diagnostic”, “iatrogenic”, “clinical”, “phlebotomy”), along with relevant synonyms. Several relevant studies were identified, including one randomized controlled trial. This trial compared a restricted blood sampling protocol with standard care in 102 extremely preterm infants and found that reducing diagnostic blood losses by one-third substantially decreased red blood cell requirements during admission. In addition, other observational studies were aimed at quantifying cumulative diagnostic blood losses in extremely preterm infants, with varying estimates reported. One single-center study reported a median total blood loss of 16.5 mL [IQR, 12.3–21.1 mL] during first 4 weeks of life in a cohort of 104 extremely preterm infants (median birth weight: 1180 g). Another single-center study found a median blood loss of 24.2 mL/kg [IQR, 15.8–30.3 mL/kg] (n = 20 extremely preterm infants, median birth weight: 835 g) over the same period. A third study in two Swedish neonatal intensive care units reported a median blood loss of 40.4 mL/kg [IQR, 23.9–53.3 mL/kg] during the first two weeks of life (n = 149 extremely preterm infants, median birth weight: 797 g).

**Added value of this study**

This is the first prospective study estimating diagnostic blood losses in extremely preterm infants on a large scale in Europe. In this cohort study of 320 infants in 22 European countries, median cumulative diagnostic blood loss at day 28 in infants born at 24, 25, 26, 27 weeks’ gestation was 21.9 mL/kg, 13.8 mL/kg, 11.7 mL/kg, 8.0 mL/kg, respectively. This corresponds to 49.6%, 25.9%, 19.7%, 11.5% calculated initial blood volume (assuming 70 mL/kg birth weight). Cumulative diagnostic blood loss by day 28 was higher among infants admitted to centers with small-volume analyzers (8.2 mL/kg), compared to infants admitted to centers with medium- and large-volume analyzers (17.3 and 19.9 mL/kg, respectively).

**Implications of all the available evidence**

These findings highlight the considerable diagnostic blood loss in extremely preterm infants during admission. This underlines the need to reduce both the frequency of diagnostic sampling and volumes required for laboratory testing where possible.

## Introduction

One main non-physiological contributor to anemia in preterm infants is iatrogenic blood loss due to repeated diagnostic sampling throughout their stay in the neonatal intensive care unit (NICU). Extremely preterm infants undergo extensive routine laboratory testing, especially in the first days after birth.^1^ However, due to the relatively small circulating blood volume, equivalent to approximately 70 mL/kg, each laboratory test requires a substantial proportion of their total blood volume.^2^, ^3^, ^4^ It is estimated that repeated phlebotomies in preterm infants can lead to a cumulative blood loss exceeding a third of the initial blood volume, as reported in several smaller studies.^1^^,^^2^^,^^5^^,^^6^

Not surprisingly, frequent diagnostic blood losses have been associated with a higher and earlier need for red blood cell (RBC) transfusions.^2^ Recent data suggest many preterm infants will require at least one RBC transfusion during neonatal intensive care.^7^ Minimizing diagnostic blood losses could thus potentially help prevent anemia of prematurity and reduce the need for RBC transfusions. However, data on cumulative blood loss resulting from laboratory testing in extremely preterm infants are limited. Additionally, little is known about variations between countries and centers, as diagnostic blood losses vary depending on protocols for routine blood tests, frequency of laboratory testing, and type of analyzers used. Therefore, we aimed to estimate diagnostic blood losses in extremely preterm infants during the first four weeks of life, and to detect variations across 22 European countries.

## Methods

### Study design

This study is a secondary outcome analysis of the International Neonatal Transfusion Point Prevalence (INSPIRE) study, a prospective, observational, cohort study describing RBC, platelet and plasma transfusion practices across 64 NICUs in 22 European countries that has been previously published.^7^, ^8^, ^9^ Countries with larger populations contributed a proportionally greater number of centers, with the aim of achieving a representative cohort. Within each country, national coordinators invited units selected to reflect the national landscape of neonatal care, considering factors such as unit size, academic versus non-academic status, and availability of surgical services. Study protocol was registered in the ISRCTN registry (ISRCTN17267090).

### Ethics

The study received approval from the Medical Ethics Committee of the Leiden University Medical Center, the Netherlands. The study was subsequently reviewed by national or regional ethical review boards in participating countries. Study conduct complied with the Declaration of Helsinki and the General Data Protection Regulation.^10^^,^^11^

### Parent advisory board

We established a parent advisory board for the study in collaboration with the Global Foundation for the Care of the Newborn Infant (GFCNI). At one of our meetings, parents expressed great concern about frequent diagnostic blood losses for laboratory testing. In response to their feedback and given the established association between diagnostic blood losses and RBC transfusions, we included diagnostic blood loss as a secondary outcome to the study.

### Participants

The study prospectively included all infants born <32 weeks gestational age (GA), who were admitted to a participating NICU during the study period, without predefined exclusion criteria. Parents/guardians provided informed consent, oral or written, if required by national or regional legislation. Number of eligible infants for whom consent was not obtained was not recorded. We restricted data collection for the current analysis to extremely preterm infants born <28 weeks GA, who were enrolled during the first 28 postnatal days (or any part thereof).

### Procedures

We collected data during a six-week study period in each participating center. Total observation period was between September 2022 and August 2023. We included infants at NICU admission, date of consent, or start of study period, whichever occurred last. We followed infants until death, discharge, or end of study period, whichever occurred first. Inherent to our dynamic cohort study design, the number of infants in study follow-up varied per day. For each infant, we collected daily counts of the following laboratory tests: full blood count, blood type screening, blood culture, blood gas, coagulation screen (APTT/PT, INR or thromboelastography), bedside glucose, bilirubin, urea and electrolytes (U&E). We requested clinicians to only record laboratory tests that required a separate blood draw, accounting for the possibility of combined testing from a single sample. For example, if glucose was measured in conjunction with a blood gas, this was not documented as a separate additional laboratory test. We defined diagnostic sampling as any method of blood sampling for diagnostic testing, irrespective of method of sampling, whether from a central or peripheral arterial line, venipuncture or capillary puncture. Additionally, for each participating center, we collected the volumes required for each test in accordance with local laboratory specifications (where applicable, we assumed the range midpoint for tests with a varying blood volume required). Lasty, we documented any events of major bleeding, culture-confirmed sepsis, necrotizing enterocolitis, invasive mechanical ventilation, or surgery between days 1–28 (see Table 1 for definitions). Local investigators were contacted at regular intervals during and after the study period to verify and complete data collection. No missing data were present in dataset for this analysis. Data on race or ethnicity were not collected due to constraints on the scope of data collection. We used a certified electronic Castor database that complies with ICH E6 Good Clinical Practice standards to collect data.

**Table 1:** Patient characteristics.

Extremely preterm infants (n = 320)	
Female sex, n (%)	147 (45.9)
Gestational age at birth, in weeks + days (median (IQR))	26 + 1 (25 + 0–27 + 1)
Birth weight, in grams (median (IQR))	800 (650–940)
Congenital anomalies, n (%)	3 (0.9)
RBC transfusion, n (%)^a^	181 (56.6)
Major bleeding, n (%)^b^	52 (16.3)
Necrotizing enterocolitis, n (%)^b^	21 (6.6)
Sepsis, n (%)^b^	62 (19.4)
Invasive mechanical ventilation, n (%)^b^	206 (64.4)
Surgery, n (%)^c^	35 (10.9)
Mortality, n (%)^a^	47 (14.7)

See Supplementary Data eTable 1 for definitions.

a During postnatal day 1–28.

b At least one episode during postnatal day 1–28.

c Any type, during postnatal day 1–28.

### Outcomes

Primary outcome measures included (1) proportion of infants with laboratory testing per day, (2) type of laboratory testing per day, (3) daily diagnostic blood loss, (4) estimated cumulative diagnostic blood loss by postnatal day 28, stratified per GA. Secondary outcome measures included (5) RBC transfusion requirements, (6) number of laboratory tests and associated diagnostic blood losses per infant on day 1 and 2, overall and stratified per center. This last outcome examined potential differences in laboratory testing patterns after birth, focusing on the first two days of life to minimize patient-mix differences later in the clinical course and facilitate comparison between centers. Centers with fewer than 2 infants in study follow-up on postnatal day 1 and 2 were omitted from this analysis. Additionally secondary outcome measures included (7) blood volume required per laboratory test per center, (8) total blood volume required for all laboratory tests combined per center, and (9) estimated cumulative diagnostic blood loss and other clinical outcomes, stratified by center analyzer volume. For this last outcome, we divided participating centers into three groups according to total blood volume required for all laboratory tests combined: centers with small-volume analyzers (lowest third), medium-volume analyzers (middle third), and large-volume analyzers (highest third).

### Statistical analyses

We formulated a statistical analysis plan prior to conducting the analyses (Supplementary 3). We estimated daily diagnostic blood losses by multiplying laboratory test counts per infant by the blood volumes required for each test at their center. We then converted these daily diagnostic blood losses from mL to mL/kg birthweight by dividing by the birthweight of the infant.

As the dynamic cohort design precluded individual longitudinal data across days 1–28 for all infants, cumulative diagnostic blood losses were estimated by summing median daily blood loss (mL/kg) per group across days 1–28. Infants were included in the analyses only for the days they remained in study follow-up, and results are therefore presented descriptively with corresponding confidence intervals rather than formal statistical comparisons.

We also expressed this as a proportion of the calculated initial blood volume, which was computed by multiplying median birthweight per GA by a blood volume of 70 mL/kg. Given varying reports of the total blood volume in extremely preterm infants, we followed the example of Hellström et al., who based this assumption on two prior publications reporting circulating volumes ranging from 62 to 78 mL/kg.^2^, ^3^, ^4^ Initial blood volumes calculated using this fixed estimate of 70 mL/kg should thus be interpreted with caution, as they represent theoretical estimates, particularly in the context of practices such as delayed cord clamping. Infants born at 22 or 23 weeks of gestation were grouped with those born at 24 weeks of gestation.

Distributions of variables were assessed graphically using histograms to inform the choice of summary statistics. Patient and center characteristics are reported as medians (interquartile ranges, IQR) or numbers (%). We conducted statistical analyses using STATA statistical Software (version 16.1, Texas, USA). We created figures using GraphPad Prism (Version 9.3.1, California, USA). This report followed the Reporting of Observational Studies in Epidemiology (STROBE) reporting guidelines.

### Role of the funding source

The funders had no role in the design and conduct of the study; collection, management, analysis, and interpretation of the data, preparation, review, or approval of the manuscript; and decision to submit the manuscript for publication.

## Results

### Patients

The INSPIRE study included a total of 1143 preterm infants, of which 320 (30%) were extremely preterm infants (GA <28 weeks) enrolled between day 1–28 (see eFig. S1 for flowchart). Their median GA at birth was 26 weeks plus 1 day (IQR, 25 weeks plus 0 days to 27 weeks plus 1 day), with a median birthweight of 800 g (650–940); 147 infants were female (46%). Patient characteristics are summarized in Table 1. A total of 181 (57%) infants received at least one RBC transfusion between day 1–28 (median number of transfusions: 2 (1–3), median total volume received: 25 mL/kg (15–45)).

### Diagnostic blood losses postnatal day 1–28

**Fig. 1:**
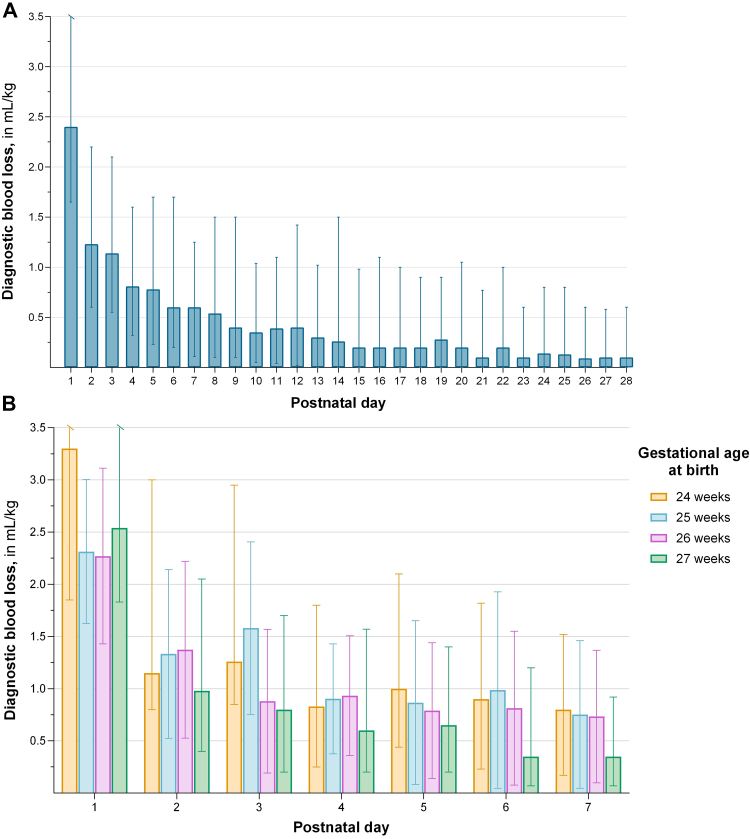
**Median diagnostic blood loss per day. A.** Median diagnostic blood loss over day 1–28. **B.** Median diagnostic blood loss over day 1–7, stratified per gestational age. | In mL/kg birthweight, whiskers represent the 25th to 75th percentile range. Univariate linear regression analysis showed that daily diagnostic blood loss (mL/kg birthweight) decreased significantly with increasing postnatal age (log-transformed) (β = −0.912 [95% CI, −0.982 to −0.842], p < 0.001).

**Fig. 2:**
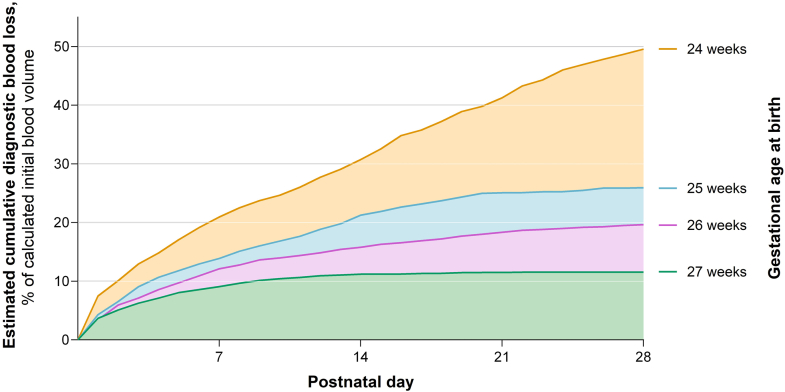
**Estimated cumulative diagnostic blood loss over postnatal day 1–28.** Calculated initial blood volumes (assuming a blood volume of 70 mL/kg birthweight (BW)) were 44.1 mL for 24 weeks (median BW: 630 g, n = 69), 53.4 mL for 25 weeks (median BW: 763 g, n = 53), 59.7 mL for 26 weeks (median BW: 853 g, n = 88), and 69.0 mL for 27 weeks (median BW: 986 g, n = 94).

The proportion of infants with at least one diagnostic sampling ranged from 97% (170/175) on day 1–58% (86/148) on day 28 (eFig. S2 in Supplementary 1). Daily diagnostic blood losses were highest in the first week of life (Fig. 1A) and decreased significantly with increasing postnatal age (log-transformed) (β = −0.912 [95% CI, −0.982 to −0.842], p < 0.001). Diagnostic blood losses during the first week of life stratified by GA are highlighted in Fig. 1B. Blood gases accounted for the largest proportion of total daily tests, with a relatively consistent distribution of test types for days other than day 1 (eFig. S3 in Supplementary 1). By day 28, median estimated cumulative diagnostic blood loss during admission was considerably higher in infants born at 24 weeks gestation (49.6% of calculated initial blood volume [IQR, 16.8–109.5]), compared to those born at 25 weeks (25.9% [IQR, 9.7–67.3]), 26 weeks (19/7% [IQR, 6.0–49.6]), or 27 weeks (11.5% [IQR, 4.5–39.5]) (Fig. 2, with data presented in mL/kg in eFig. S4).

### Variation across centers

**Fig. 3:**
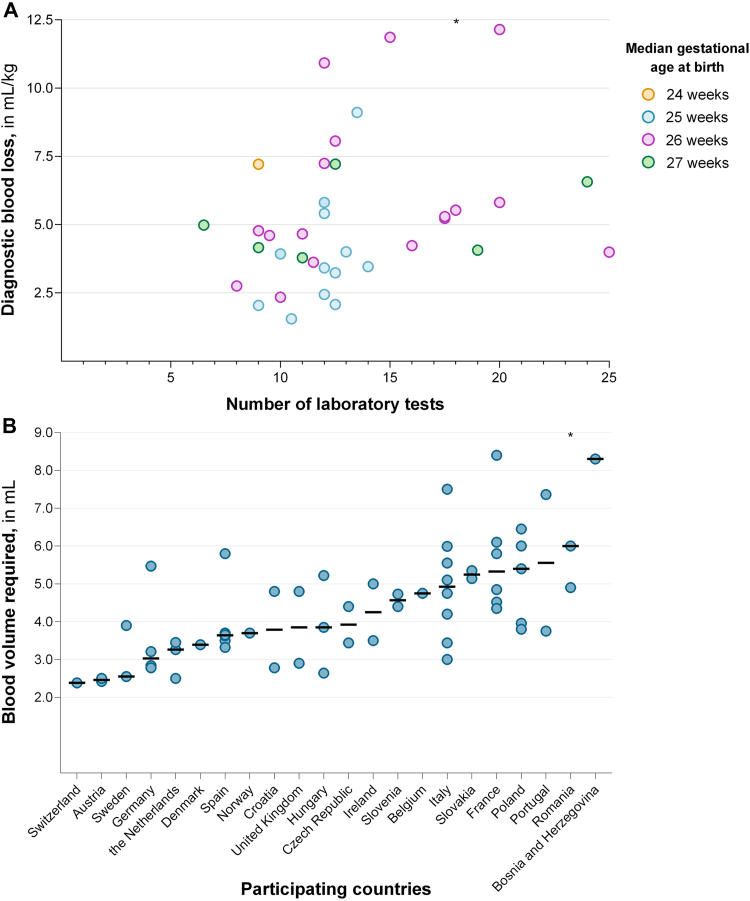
**Variation between centers. A.** Median number of laboratory tests and total diagnostic blood losses on postnatal day 1 and 2 per center | Based on 151 infants from 37 centers. Centers with fewer than 2 infants in study follow-up on postnatal day 1 and 2 were omitted from the plot (n = 12). ∗Datapoint outside Y-axis (median diagnostic blood loss: 26.7 mL/kg). **B.** Total blood volume required for all laboratory tests combined | Total volume needed for a full blood count, a blood type screening, a blood culture, a blood gas, a coagulation screen, a bedside glucose, a bilirubin, and an urea and electrolytes. Dots represent the participating centers, horizontal bars indicate median total blood volume required per country. ∗Datapoint outside Y-axis limit (blood volume: 14.7 mL).

A total of 163 (51%) infants were followed on both postnatal day 1 and 2. eFig. S5 shows the total number of laboratory tests and total diagnostic blood loss for day 1 and 2 combined, highlighting individual variation. In Fig. 3A, these data are presented stratified by center, with varying laboratory testing patterns observed. Some centers performed fewer tests but required more blood, whereas others conducted more tests with less associated diagnostic blood loss. Median total number of laboratory tests performed on day 1 and 2 combined ranged from 6.5 to 25 tests per center, and median associated diagnostic blood losses ranged from 1.6 to 26.7 mL/kg between centers. Across all participating centers (n = 64), median blood volumes required per laboratory test were 0.5 mL (IQR, 0.3–0.5) for a full blood count, 0.5 mL (IQR, 0.3–1.0) for a blood type screening, 1.0 mL (IQR, 1.0–1.0) for a blood culture, 0.2 mL (IQR, 0.1–0.3) for a blood gas, 1.0 mL (IQR, 0.5–1.4) for a coagulation screen, 0.05 mL (0.02–0.10) for a bedside glucose, 0.2 mL (IQR, 0.1–0.5) for a bilirubin concentration, and 0.5 mL (IQR, 0.4–0.8) for a U&E. Fig. 3B highlights the total blood volume required in a hypothetical scenario where clinicians order each test once: full blood count, blood type screening, blood culture, blood gas, coagulation screen, bedside glucose, bilirubin level, and U&E. Total blood volume for all tests combined ranged markedly from 2.4 to 14.7 mL across centers, with a median of 4.1 mL (IQR, 3.4–5.3 mL).

### Comparison between centers with small, medium, and large-volume analyzers

Infants admitted to participating centers with small-volume analyzers experienced half the cumulative diagnostic blood loss by day 28 (8.2 mL/kg [IQR, 3.2–20.3]), compared to those admitted to centers with medium- and large-volume analyzers (17.3 mL/kg [IQR, 4.7–43.3], and 19.9 mL/kg [6.4–52.8], respectively). However, transfusion rates and total transfusion volumes were similar among the three groups, as were other clinical outcomes (Table 2).

**Table 2:** Estimated cumulative diagnostic blood loss at day 28 and clinical outcomes, stratified by analyzer volume.

	Infants admitted to centers with small-volume analyzers (≤3.5 mL^a^) n = 137	Infants admitted to centers with medium-volume analyzers (>3.5 to ≤4.85 mL^a^) n = 87	Infants admitted to centers with large-volume analyzers (>4.85 mL^a^) n = 96	p
Female sex, n (%)	64 (47)	40 (46)	43 (45)	0.96
Gestational age at birth, in weeks + days (median (IQR))	26 + 0 (24 + 6–27 + 0)	26 + 1 (25 + 1–27 + 2)	26 + 1 (25 + 0–27 + 1)	0.78
Birthweight, in grams (median (IQR))	815 (655–960)	770 (625–900)	800 (650–940)	0.39
Estimated cumulative diagnostic blood loss at day 7, in mL/kg birthweight (median (IQR))^b^	5.5 (2.9–9.0)	10.3 (4.5–17.2)	11.1 (5.5–20.4)	
Estimated cumulative diagnostic blood loss at day 28, in mL/kg birthweight (median (IQR))^b^	8.2 (3.2–20.3)	17.3 (4.7–43.3)	19.9 (6.4–52.8)	
At least 1 RBC transfusion during day 1–7, n (%)	43 (31)	27 (31)	26 (27)	0.48
At least 1 RBC transfusion during day 1–28, n (%)	76 (55)	46 (53)	59 (61)	0.76
Total RBC volume transfused, in mL/kg (median (IQR))^c^	23.1 (15.0–40.0)	30.0 (15.0–47.5)	20.0 (15.0–45.0)	0.76
Pretransfusion Hb, in g/L (median (IQR))^c^	100.5 (90.0–110.0)	100.0 (91.8–108.5)	95.0 (89.0–102.1)	0.18
Major bleeding, n (%)^d^	29 (14)	12 (14)	21 (22)	0.20
Necrotizing enterocolitis, n (%)^d^	10 (7)	7 (8)	4 (4)	0.51
Mortality, n (%)^d^	22 (16)	12 (14)	13 (14)	0.84

See Supplementary Data eTable 1 for definitions.

a Total volume needed for a full blood count, a blood type screening, a blood culture, a blood gas, a coagulation screen, a bedside glucose, a bilirubin, and an urea and electrolytes.

b Cumulative diagnostic blood losses were estimated by summing median daily blood loss (mL/kg) per group across days 1–28, results are therefore presented descriptively with corresponding confidence intervals.

c Among those receiving at least one RBC transfusion, during postnatal day 1–28.

d During postnatal day 1–28. p-values were calculated using Pearson’s chi-square test for categorical variables and the Kruskal–Wallis test for continuous variables, a p-value < 0.05 was considered to be statistically significant.

## Discussion

In this European prospective study, we found that extremely preterm infants experienced significant diagnostic blood loss during neonatal intensive care, particularly in infants born at 24 weeks with cumulative diagnostic blood loss accounting for half of their calculated initial blood volume. Importantly, frequency of laboratory testing after birth and analyzer volumes needed for diagnostic sampling varied markedly across centers. Cumulative blood loss in infants admitted to centers with medium- or large-volume analyzers was twice as large compared to those admitted to centers with small-volume analyzers.

Our findings are consistent with smaller, national studies that have previously described diagnostic blood loss in extremely preterm infants, but with varying estimates.^1^^,^^2^^,^^6^^,^^12^^,^^13^ Similarly to these results, we observed highest daily diagnostic blood losses in the first week of life. This may partly be explained by the fact that extremely preterm infants often require respiratory support after birth, which necessitates frequent blood gas testing. Additionally, their immature physiology and hemodynamic instability can require close monitoring of laboratory parameters, for which the need may decrease over time as infants stabilize.

Proportionate to blood volume, estimated cumulative blood loss over day 1–28 in infants born at 24 weeks was approximately twice as high compared to those born at 25–27 weeks gestation. This is likely because of their smaller initial circulating volume (due to their lower birthweight) combined with a higher need for laboratory testing. Additionally, volumes required for laboratory testing remain constant regardless of gestational age, meaning that each test has a relatively greater impact on infants born at 24 weeks compared to those born at later gestational ages. These results underscore the importance of patient blood management in this particularly vulnerable group and the need for careful evaluation of each laboratory test.^14^^,^^15^ However, in the context of extreme prematurity, some degree of diagnostic blood loss is likely unavoidable.

We observed striking differences in blood volumes required for similar laboratory tests across participating centers. Notably, this resulted in higher cumulative diagnostic blood loss among infants cared for in centers with medium- to large volume analyzers compared to those admitted to centers with small-volume analyzers. Our findings highlight the potential of micro-analyzers to substantially decrease overall diagnostic blood loss. However, we did not observe a lower RBC transfusion rate among infants cared for in centers using small-volume analyzers. The proportion of infants receiving at least one transfusion was similar to that of centers using medium to large volume analyzers, possibly because our study was not powered to assess this. Additionally, transfusion decisions are likely to be multifactorial and not only driven by diagnostic blood loss, but also by the transfusion thresholds applied, the infant’s clinical condition, local awareness for anemia management, and availability of resources. In a large RCT among adults, the simple measure of reducing sample volumes has been shown to decrease transfusion rates.^16^ In absence of a similar RCT among preterm infants, it remains unknown whether using of micro-analyzers could meaningfully reduce transfusion requirements in this population. A retrospective study among preterm infants found that replacing conventional blood gas analyzers with a bedside point-of-care analyzer requiring a smaller blood volume was associated with a significant reduction in the mean number of RBC transfusions administered^17^ Similarly, another study among preterm infants compared RBC transfusion rates before and after implementing three laboratory analyzers that required lower blood collection volumes and found that the number of transfusions and transfusion volumes decreased significantly.^18^ Although acquiring these micro-analyzers may be costly and formal cost-effectiveness analyses are lacking, their use may lead to long-term cost savings by reducing diagnostic blood losses and lowering the number of RBC transfusions required.

We also found the number of laboratory tests performed after birth varied across centers. The type of analyzers available in a center may also play a role in these patterns, as the amount of blood required may influence the decision to order a laboratory test. Additionally, differences in patient illness severity may also have contributed to the observed variation. Other potential reasons for differences between centers could be related to resource availability or local awareness for diagnostic blood losses. Some centers with fewer laboratory tests could also use strict protocols for diagnostic sampling during admission. This strategy has been found to significantly reduce diagnostic sampling loss and RBC requirements during NICU admission, as demonstrated in an RCT among preterm infants comparing a restricted sampling protocol with standard practice.^19^ Importantly, the observed differences should not be interpreted as indicators of quality of care, but rather as reflections of these previously described aspects.

Although laboratory testing is an essential part of intensive care, there are several arguments for reducing diagnostic blood loss in preterm infants. Firstly, preterm infants are known to have reduced ability to increase endogenous erythropoietin production in response to anemia and tissue hypoxia compared to older children or adults and are thus less able to compensate for severe diagnostic blood loss.^20^^,^^21^ Secondly, there is a need to reduce the number of diagnostic punctures given the long-term effects of painful procedures in preterm infants.^22^^,^^23^ One study found that neonates underwent a median of 47 punctures for diagnostic sampling during the first 28 days of life, and each puncture is associated with a painful stimulus.^1^^,^^24^ The use of central or peripheral lines may help to reduce the number of painful events. A study among extremely preterm infants showed that a median of nine blood samples could be taken from a single peripheral arterial catheter, substantially reducing the need for repeated punctures.^25^ However, although use of a central line avoids the need for diagnostic sampling punctures, this may also contribute to a more liberal decision-making for laboratory testing.

Thirdly, previous studies have shown that diagnostic blood losses during the first month of life approximated RBC transfusion volumes received by preterm infants during this period.^1^^,^^2^^,^^26^ Decreasing the amount and frequency of diagnostic sampling may thus be an effective strategy to reduce the need for RBC transfusions, as these are given to compensate diagnostic blood loss.^12^ Lastly, RBCs of preterm infants carry predominantly fetal hemoglobin (HbF), which has a higher oxygen affinity than adult hemoglobin (HbA). Frequent diagnostic sampling, combined with transfusions of adult RBCs to offset these losses, contribute to a progressive depletion of HbF levels, which has been associated with retinopathy of prematurity and bronchopulmonary dysplasia.^27^

Another promising strategy to reduce diagnostic blood losses is obtaining initial laboratory tests after birth from umbilical cord blood (UCB).^28^ This is particularly important since the largest volume of diagnostic blood loss occurs within the first day of life. Previous studies have suggested that results for tests like a full blood count, C-reactive protein and blood culture are comparable to those collected directly from the infant.^29^, ^30^, ^31^ Two RCTs involving preterm infants found that using UCB for diagnostic sampling at admission significantly delayed time to first transfusion.^32^^,^^33^ Additionally, complementary to using UCB for diagnostic sampling, delayed cord clamping is an effective strategy to increase the circulating blood volume at birth, which helps to compensate for diagnostic blood losses during the NICU stay.^34^, ^35^, ^36^

Additionally, non-invasive monitoring methods, such as transcutaneous measurements of pO_2_ and pCO_2_ or end-tidal CO_2_ measurements, may minimize the need for laboratory testing during mechanical ventilation or in critically ill infants.^37^, ^38^, ^39^ Moreover, other strategies may include continuous glucose monitoring using a subcutaneous sensor, which has been validated for use in preterm infants and was found to significantly decrease the number of blood samples required per infant.^40^^,^^41^

Our study has several limitations. Despite the significant diagnostic blood loss observed, our findings probably underestimate true blood loss. Additional blood loss is very likely, as we did not account for hidden blood loss, such as blood in gauze pads or discarded blood volumes resulting from overdraw. A previous study found that the blood volumes drawn for diagnostic sampling exceeded the required volume by a mean of 19% (SD: 1.8%), highlighting the potential magnitude of this issue.^42^ We also limited our data collection to the first 28 postnatal days of life and the most common laboratory tests and did not include other diagnostic tests, which may have further contributed to an underestimation of true blood loss. Secondly, we did not record the method of sampling (either capillary, venous, arterial, or via a central line) and if infants required a direct needle puncture for the diagnostic sampling. This data could have provided further insights into the burden of laboratory testing on these infants and potential patterns between collection methods and diagnostic testing. Lastly, the small number of infants per country did not allow for adjustments of patient-mix, which could explain part of the observed variations.

In summary, we found evidence of significant diagnostic blood losses in extremely preterm infants during the first 28 days of life in neonatal intensive care. We observed considerable variation between European centers in blood volumes required for laboratory testing. Infants cared for in centers with medium- or large-volume analyzers experienced higher cumulative diagnostic blood loss. Our findings highlight the need to reduce both the frequency of diagnostic sampling and volumes required for laboratory testing. Furthermore, a better understanding of what constitutes ‘appropriate’ or ‘inappropriate’ laboratory testing in extremely preterm infants is needed to inform future strategies to minimize diagnostic blood losses, thereby potentially preventing iatrogenic anemia and reducing need for RBC transfusions.

## Contributors

The members of the INSPIRE Study Group are listed in Supplementary 2. All individuals included in the Study Group contributed as local investigators for their respective NICUs.

*Concept and design:* Houben, Fustolo-Gunnink, Fijnvandraat, New, Stanworth, Deschmann, Roehr, Dame, van der Bom, Lopriore.

*Acquisition, analysis, or interpretation of data:* Houben, Fustolo-Gunnink, Fijnvandraat, Caram-Deelder, Aguar Carrascosa, Beuchée, Brække, Cardona, Debeer, Domingues, Ghirardello, Grizelj, Hadžimuratović, Heiring, Lozar Krivec, Malý, Matasova, Moore, Mühlbacher, Szabó, Szczapa, Zaharie, de Jager, Reibel, Deschman, Roehr, Dame, le Cessie, van der Bom, Lopriore.

*Drafting of the manuscript:* Houben, Fustolo-Gunnink, Fijnvandraat, Lopriore.

*Critical revision of the manuscript for important intellectual content:* All authors read and approved the final version.

*Statistical analysis:* Houben, Caram-Deelder, le Cessie, van der Bom.

*Obtained funding:* Fustolo-Gunnink.

*Administrative, technical or material support:* Houben, Caram-Deelder, Aguar Carrascosa, Beuchée, Brække, Cardona, Debeer, Domingues, Ghirardello, Grizelj, Hadžimuratović, Heiring, Lozar Krivec, Malý, Matasova, Moore, Mühlbacher, Szabó, Szczapa, Zaharie, de Jager, Reibel, Deschman, Roehr, Dame.

*Supervision:* Fustolo-Gunnink, Fijnvandraat, van der Bom, Lopriore.

Houben and Caram-Deelder have directly accessed and verified the underlying data reported in the manuscript.

## Data sharing statement

No consent was obtained for further data sharing. External applications for data access will therefore only be taken into consideration after the prior written consent of all participating centers.

## Declaration of interests

Fustolo-Gunnink disclosed receiving grants from Sanquin Blood Supply Foundation (PPOC21-08/L2588, RES/00264), the European Blood Alliance (EBA Grant Agreement 2021-02), and the European Society for Paediatric Research (ESPR Post-Doc Research Grant 2020). Fustolo-Gunnink disclosed her role as Chair of the Special Interest Group on Haematology of the European Society for Pediatric Research. Muehlbacher disclosed receiving compensation from Sanquin Blood Supply Foundation. Szczapa disclosed his role as President of the Polish Neonatal Society and his role as National Consultant for Neonatology in Poland. All other authors have no conflicts.
