# Supplementary material for: Blood loss due to diagnostic testing in extremely preterm infants in 22 European countries: a prospective observational study
Source: eClinicalMedicine. 2026 Jun 26;97:104035. doi: 10.1016/j.eclinm.2026.104035 (PMC13324306; doi:10.1016/j.eclinm.2026.104035)
Supplement: Supplementary 1 [file mmc1.pdf]

Supplement 1. eTables and eFigures

eTable 1. Definitions

eFigure 1. Study flowchart

eFigure 2. Proportion of infants with at least one phlebotomy over day 1-28

eFigure 3. Distribution of laboratory test types over day 1-28 (100% stacked bar chart)

eFigure 4. Estimated cumulative phlebotomy loss (in mL/kg) over postnatal day 1-28

eFigure 5. Laboratory tests and diagnostic blood losses on postnatal day 1 and 2

eTable 1. Definitions

**Event**	**Definitions**
**Major congenital anomalies**	Chromosomal anomalies, syndromes likely affecting long-term outcome, major malformations requiring surgical correction during newborn period, or cyanotic heart defects
**Bleeding disorder**	Any genetic or congenital disorders related to a higher risk of bleeding
**Major bleeding**	Any of the following bleedings:<br>– Intraventricular hemorrhage (IVH) is defined as IVH Grade 3 (extension of bleeding involving >50% of ventricular area or dilation of ventricle) or IVH Grade 4/IPE (extension of bleeding into surrounding parenchyma) (1)<br>– Intracranial hemorrhage (non-IVH) is defined as a major bleeding if any of the following apply: neurosurgical intervention is required; radiological imaging showing a midline shift; clinical signs and symptoms of an oxygen deficit with significant derangement of laboratory investigations<br>– Pulmonary bleeding is defined as acute fresh blood through the endotracheal tube associated with increased ventilatory requirements or the need for intubation and ventilation<br>– Frank rectal bleeding is defined as macroscopic fecal bleed (not if only occult positive)
**Sepsis**	Culture-positive sepsis
**NEC**	At least NEC Stage III according to the Modified Bell Staging Criteria (2)
**Invasive mechanical ventilation**	Any form of invasive respiratory support for which the neonate is intubated, including conventional mechanical ventilation and high frequency oscillation (HFO).

eFigure 1. Study flowchart

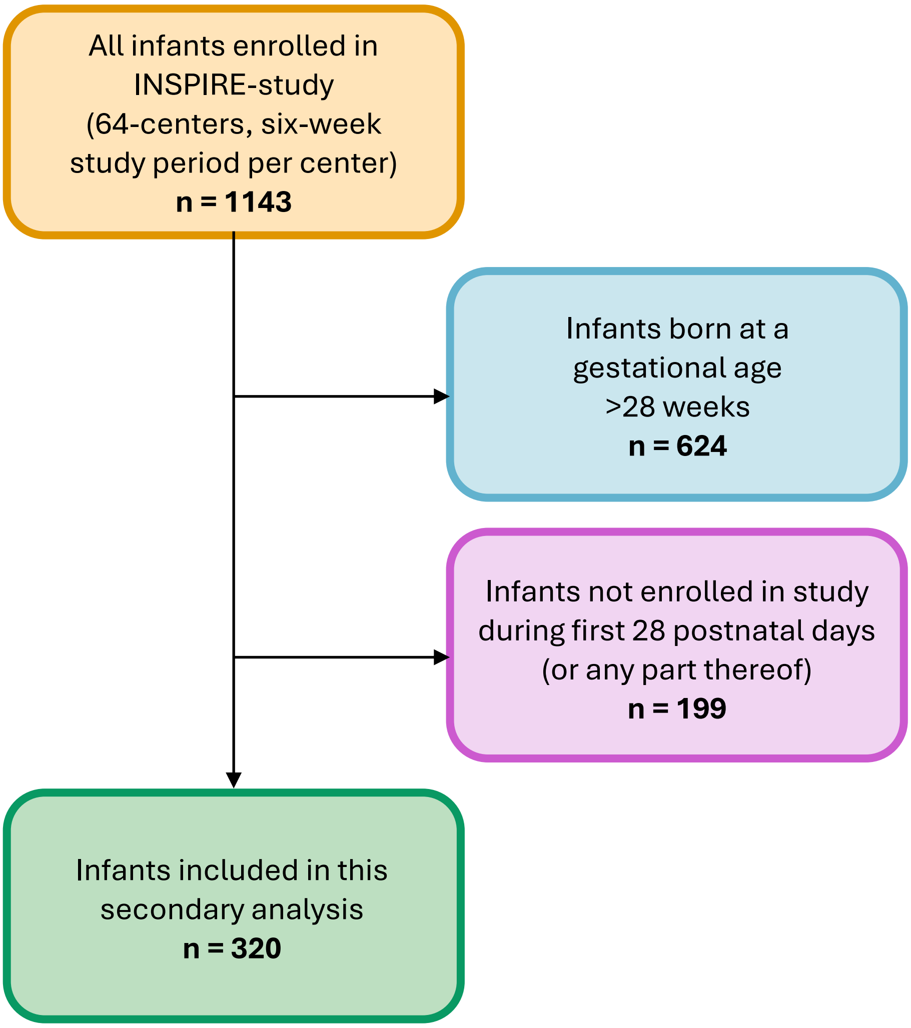

eFigure 2. Proportion of infants with at least one phlebotomy over day 1-28

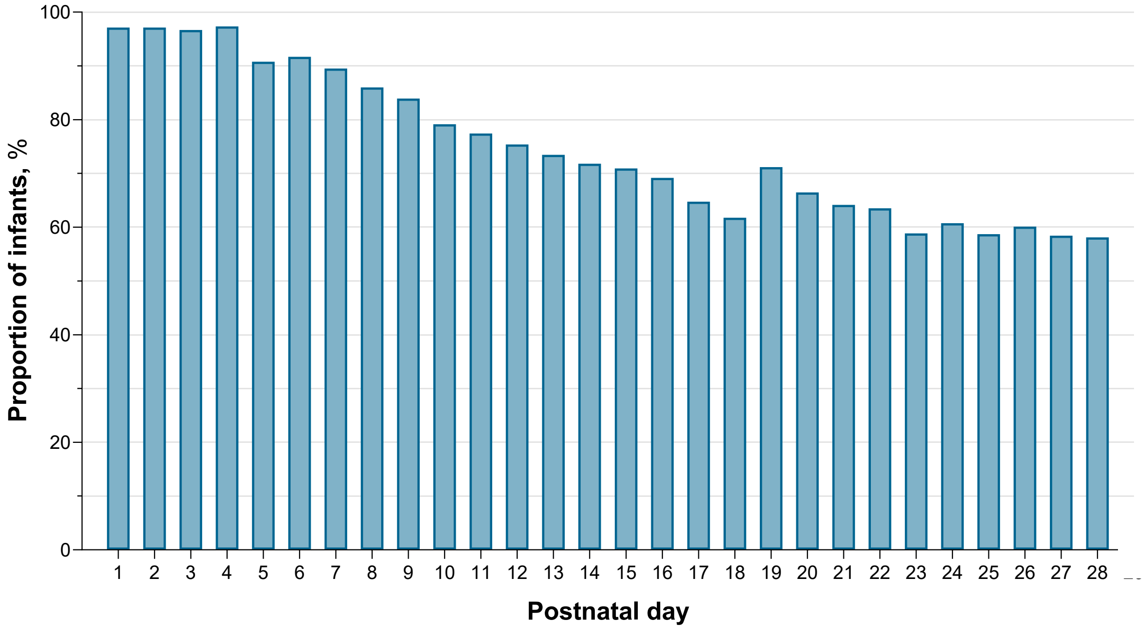

eFigure 3. Distribution of laboratory test types over day 1-28 (100% stacked bar chart)

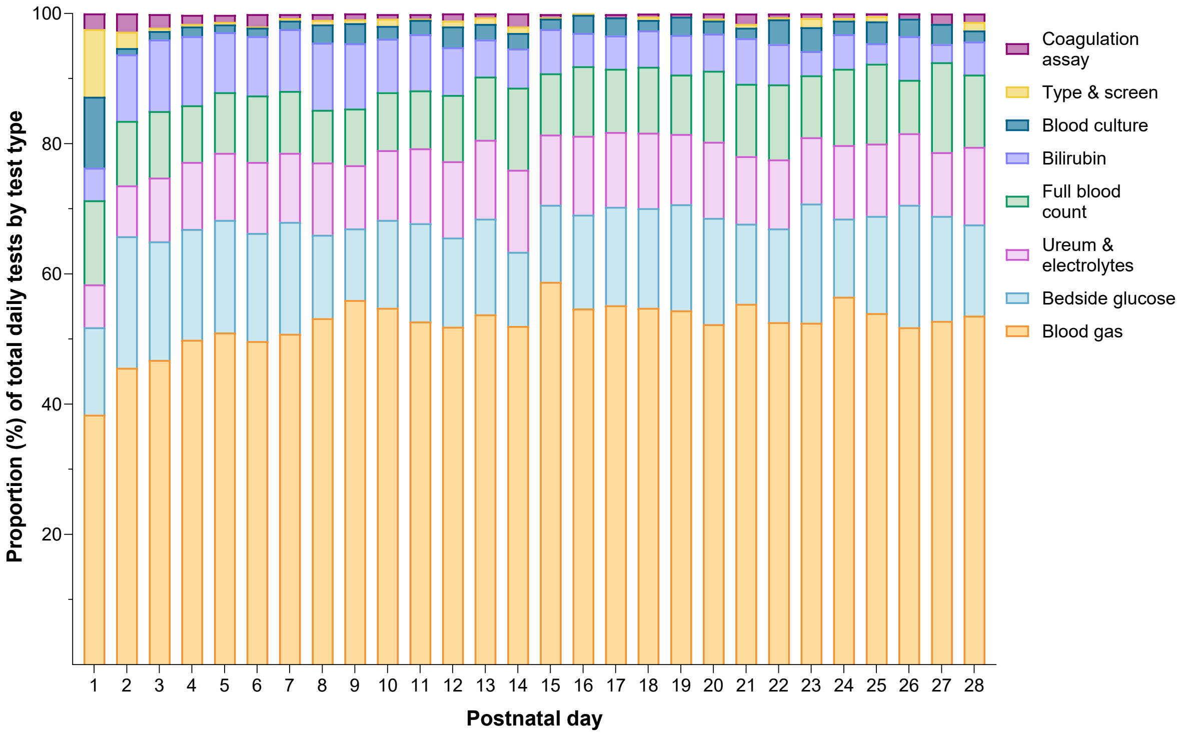

eFigure 4. Estimated cumulative phlebotomy loss (in mL/kg) over postnatal day 1-28

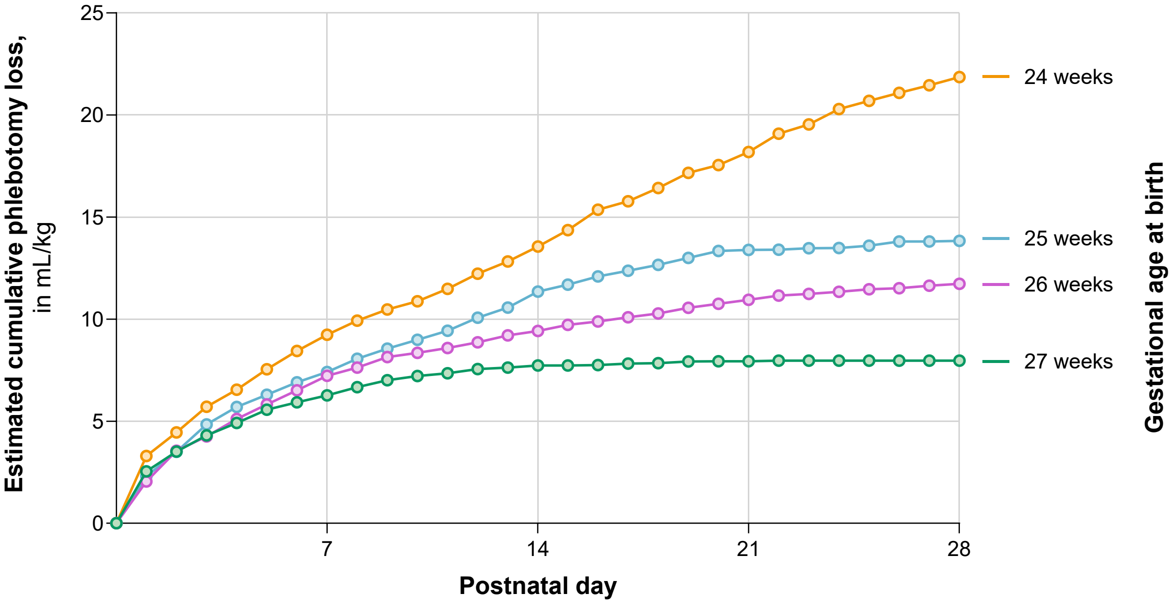

For each postnatal day, we calculated the median phlebotomy loss per gestational age. Median daily phlebotomy losses per gestational age were summed to estimate the cumulative phlebotomy loss. Median cumulative diagnostic blood loss at day 28 in infants born at 24, 25, 26, 27 weeks' gestation was 21.9 mL/kg [IQR, 7.4 – 48.3], 13.8 mL/kg [IQR, 5.2 – 35.9], 11.7 mL/kg [IQR, 3.6 – 29.6], 8.0 mL/kg [IQR, 3.1 – 27.3].

eFigure 5. Laboratory tests and diagnostic blood losses on postnatal day 1 and 2

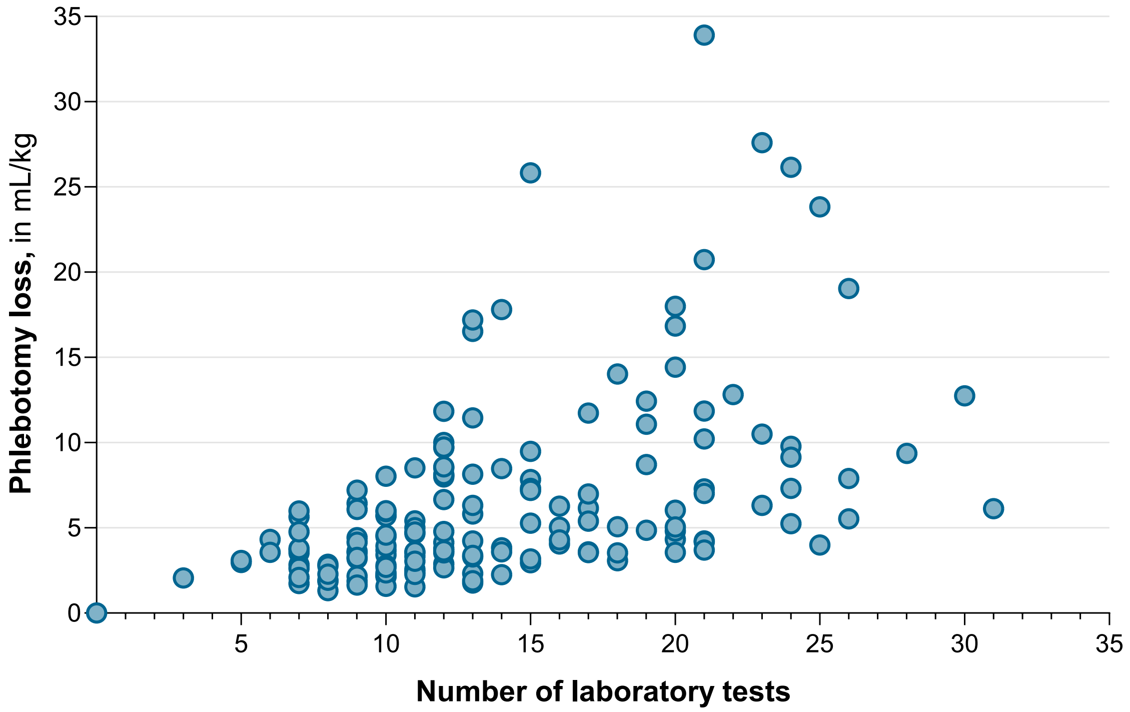

Number of laboratory tests and total diagnostic blood losses on postnatal day 1 and 2 per infant , for 163 infants in study follow-up on both these days. One datapoint outside the X-axis limit (53 tests, 10.4 ml/kg diagnostic blood loss).

References

1. Papile LA, Burstein J, Burstein R, Koffler H. Incidence and evolution of subependymal and intraventricular hemorrhage: a study of infants with birth weights less than 1,500 gm. J Pediatr. Apr 1978;92(4):529-34. doi:10.1016/s0022-3476(78)80282-0

2. Bell MJ. Neonatal necrotizing enterocolitis. N Engl J Med. Feb 2 1978;298(5):281-2.
