# Supplementary material for: Blood loss due to diagnostic testing in extremely preterm infants in 22 European countries: a prospective observational study
Source: eClinicalMedicine. 2026 Jun 26;97:104035. doi: 10.1016/j.eclinm.2026.104035 (PMC13324306; doi:10.1016/j.eclinm.2026.104035)
Supplement: Supplementary 3 [file mmc3.pdf]

STATISTICAL ANALYSIS PLAN

Iatrogenic Blood Loss Paper

Version 1.2
November 12th, 2024

Principal investigators:
Prof. Dr. E. Lopriore
Prof. Dr. J.G. van der Bom
Prof. Dr. C.J. Fijnvandraat
Dr. S.F. Fustolo-Gunnink
Prof. Dr. Christof Dame
Dr. Charles Roehr
Dr. Emöke Deschmann
Prof. Dr. Simon Stanworth
Dr. Helen New
Dr. Camila Caram Deelder

Biostatistician:
Prof. dr. Le Cessie

Data-manager:
Dr. Camila Caram Deelder

Coordinating investigator:
N.A.M. Houben, PhD student

ISRCTN registry:
Registration number ISRCTN17267090

TABLE OF CONTENTS

1. STUDY SUMMARY 3

2. STUDY OUCOME MEASURES FOR THIS PAPER 4

2.1 Study outcome measures 4

2. DATA COLLECTION 5

2.1 Patients 5

2.2 Laboratory tests 5

2.3 General remarks 5

3. DATA CLEANING 6

3.1 Data cleaning during study follow-up 6

3.2 Data cleaning after completion study follow-up 6

4. DATA ANALYSES 7

4.1 Missing data 7

4.2 Data transformation 8

4.3 General remarks 8

4.4 Patient characteristics 9

5. ANALYSIS OF STUDY OUTCOME MEASURES 10

5.1 Proportion of infants with laboratory testing per postnatal day 10

5.2 Blood volume withdrawn per postnatal day 11

5.3 Cumulative blood volume withdrawn from postnatal day 1 to 28 12

5.4 Number of laboratory tests and blood volume withdrawn on day 1 and 2 13

5.5 Blood volume required per type of laboratory test 14

5.6 Total blood volume required for all laboratory tests combined 15

6. STATEMENT 16

7. REFERENCES 17

1. STUDY SUMMARY

Study rationale: Premature infants are a highly transfused group, though robust evidence supporting neonatal transfusion practice is scarce. Red blood cell transfusions are commonly administered for neonatal anaemia, which can be caused by iatrogenic blood loss from frequent laboratory testing. Detailed contemporary data on neonatal transfusion practices in Europe, including data on iatrogenic blood loss, are lacking.

Study population: preterm infants born below 32 weeks gestation admitted to a tertiary level Neonatal Intensive Care Unit (NICU).

Study design: prospective, international, multicentre, observational study.

Study data collection: data collection took place from September 2022 to August 2023. All participating centers collected data during a fixed six-week study period. Local sites documented transfusion use in all infants in their NICU during these weeks, including infants already admitted at the start of the study period or newly admitted during the study period. Consequently, not all infants were followed from birth, and the duration of study follow-up varied, with a maximum follow-up of 42 days per included patient.

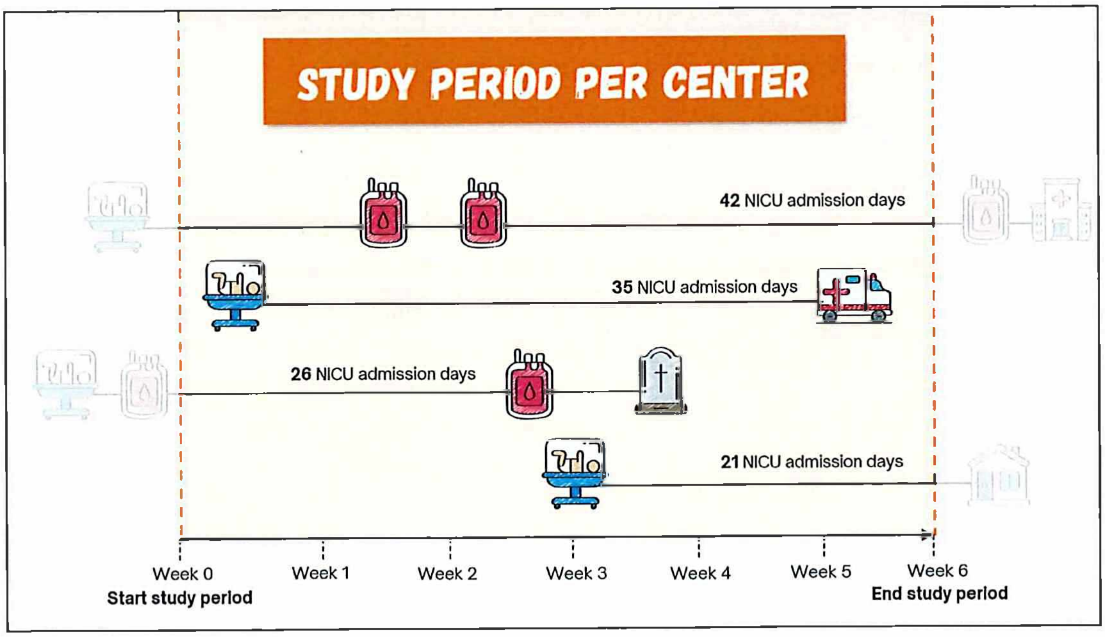

Infographic was made using images of Juicy_Fish from FlatIcon.

2. STUDY OUCOME MEASURES FOR THIS PAPER

We aim to publish multiple papers with the study data. ***This SAP only focuses on iatrogenic blood loss as part of the RBC data.***

Of note, we established a study parent advisory board (PAB) in collaboration with the European Foundation for the Care of the Newborn Infant (EFCNI). The board, consisting of parents, expressed significant concern about phlebotomy losses in preterm infants. In response to their feedback, we added the study outcome of iatrogenic blood loss to the protocol.

However, we recognize that our dynamic cohort study design does not allow for any causal conclusions on the relationship between the volume withdrawn for laboratory testing and the volume transfused. Therefore, this paper will focus on several descriptive outcomes related to iatrogenic blood loss and highlight variation between coutnries.

2.1 Study outcome measures

1. Proportion of infants with laboratory testing per postnatal day
2. Blood volume withdrawn per infant per postnatal day
3. Cumulative blood volume withdrawn from postnatal day 1 to 28
4. Volume withdrawn and number of laboratory tests on day 1 and 2
5. Blood volume required per laboratory test, per center
6. Total blood volume required for all laboratory tests combined, per center

2. DATA COLLECTION

2.1 Patients

Given the extensive study load for local investigators to collect data on laboratory tests, we decided in advance to restrict this analysis to extremely preterm infants (gestational age (GA) at birth below 28 weeks) that were followed in the study during the first 28 postnatal days (or part thereof). Extremely preterm infants are at higher risk for iatrogenic anaemia due to their relatively smaller circulating blood volume compared to older preterm infants, and most of the laboratory testing is performed in the first weeks after birth. (1, 2)

2.2 Laboratory tests

We also decided to only collected data for the following laboratory tests:

- Full blood count
- Blood type screening
- Blood culture
- Blood gas
- Coagulation assay (APTT/PT, INR or thromboelastography)
- Bedside glucose
- Bilirubin
- Urea and electrolytes (U&E)

For each laboratory test, we collected the following data:

- Required blood volume for this laboratory test, per center
- Number of tests withdrawn, per infant per postnatal day

Of note, *we requested clinicals to only record laboratory tests for which a separate blood draw was withdrawn. Example: if glucose is standardly included in your blood gas test results, it is not necessary to record this separately under 'Bedside glucose'. However, if a glucose was withdrawn independently from the blood gas, then please record that as a bedside glucose.*

2.3 General remarks

We collected all study data using a certified electronic Castor database that complies with ICH E6 Good Clinical Practice standards. Using data dependencies based on GA at birth and postnatal age of study inclusion, Castor automatically calculated whether the blood test section needed to be completed.

3. DATA CLEANING

3.1 Data cleaning during study follow-up

Center specific patient data was checked and cleaned after the completion of the study period in the respective participating center. This data quality check was used to detect outliers and impossible combinations of data entry (e.g. non-chronological dates, transfusions after end of study follow-up, etc.). Additionally, we checked for missing data in the baseline characteristics and outcome data. In case of missing data or ambiguities, the local investigator was contacted, and incorrect values were directly corrected in the data management system Castor.

3.2 Data cleaning after completion study follow-up

A general final data quality check was performed after completion of the study period in all participating centers. Possible ambiguities that were overlooked during the center specific check will be corrected, where any modifications made will be recorded in the data cleaning code.

4. DATA ANALYSES

4.1 Missing data

Missing values were limited in the eCRF since most of the collected variables were marked as mandatory to fill in. Therefore, we expect very few missing values in the dataset.

We decided in advance how to handle missing data for the following variables:

Variables	Commentary
**Number of laboratory tests per postnatal day**	The tabular format in the eCRF lacks automatic data validations for registering the number of laboratory tests per postnatal day. These tables only appear if laboratory tests were indicated on the preceding question. Clinicians can select numbers 0-10 or "Unknown" from dropdown menus for each test type per day. Blank fields will be imputed as '0' assuming clinicians most likely leave them blank to save time when tests weren't withdrawn.
**Any blood tests performed per postnatal day**	If clinicians indicated "Yes" in the question whether any blood tests were performed on a specific postnatal day, but no blood tests were selected in the dropdown menus, we will impute the preceding question to "No". Additionally, if "Unknown" we will assume that no laboratory testing is performed.

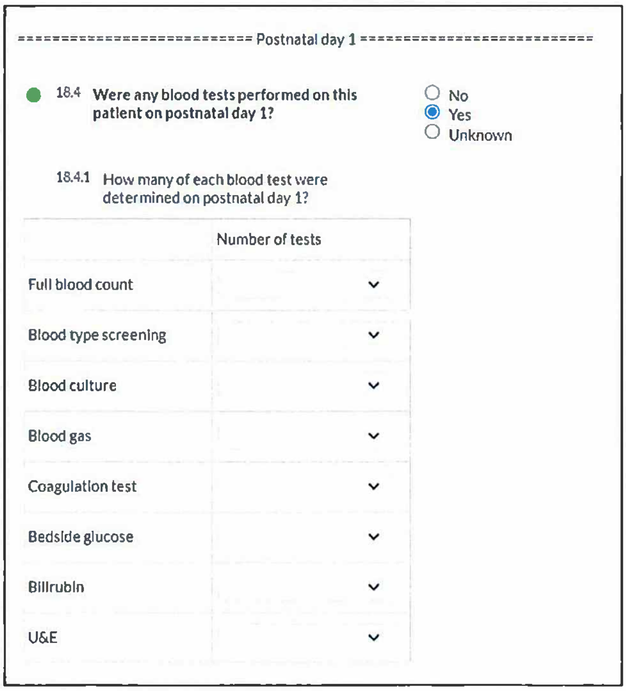
========================= Postnatal day 1 =========================

18.4 Were any blood tests performed on this patient on postnatal day 1? ○ No ◉ Yes ○ Unknown

18.4.1 How many of each blood test were determined on postnatal day 1?

	Number of tests
Full blood count	
Blood type screening	
Blood culture	
Blood gas	
Coagulation test	
Bedside glucose	
Bilirubin	
U&E	

Images: Tabular format in eCRF

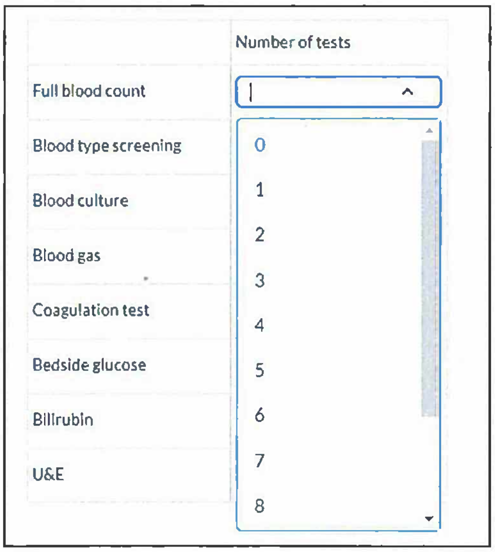

	Number of tests
Full blood count	
Blood type screening	0
Blood culture	1
Blood gas	2
Coagulation test	3
Bedside glucose	4
Bilirubin	5
U&E	6

7
8

For all other variables, the total number of missing values will be reported, with the number of infants who had one or more missing values for the variable.

4.2 Data transformation

In the center database, all participating centers recorded the volume required per laboratory test. All volumes were converted from microliters to milliliters where applicable. Center data on the required blood volumes were then linked to the respective patient data in the patient database. We calculated the total blood volume withdrawn on each postnatal day per infant by multiplying the number of tests conducted with the respective required blood volume. Where applicable, we converted the blood volume withdrawn (in mL) to mL/kg birthweight by dividing the volume withdrawn per infant by their birthweight.

4.3 General remarks

All statistical analyses will be performed using STATA statistical Software (Version 16.1, Texas, USA). Continuous data will be presented as mean (standard deviation (SD)) or as median (interquartile range (IQR)), where appropriate.

4.4 Patient characteristics

The following descriptive data on patient and center characteristics will be presented.

Patient characteristics

- Number of patients
- Sex, % female
- GA at birth, in days
- Birth weight, in grams
- Multifetal pregnancy, % singleton
- Patients with congenital anomalies, %
- Patients with at least one major bleeding*, %
- Patients with at least one NEC episode*, %
- Patients with at least one sepsis episode*, %
- Patients with at least one day of mechanical ventilation*, %
- Patients with at least one surgical procedure*, %

**During postnatal day 1 to 28 or any part thereof in the study follow-up*

Center characteristics

- Participating centers, n (% of total
- Centers with academic status, n (%)
- Centers that perform NEC surgery, n (%)
- Large centers, n (%)

5. ANALYSIS OF STUDY OUTCOME MEASURES

5.1 Proportion of infants with laboratory testing per postnatal day

We will calculate the proportion of infants with and without laboratory testing per postnatal day. Infants will be included in the analysis for the postnatal days in which they were followed in study follow-up (from day 1 to 28, or part thereof).

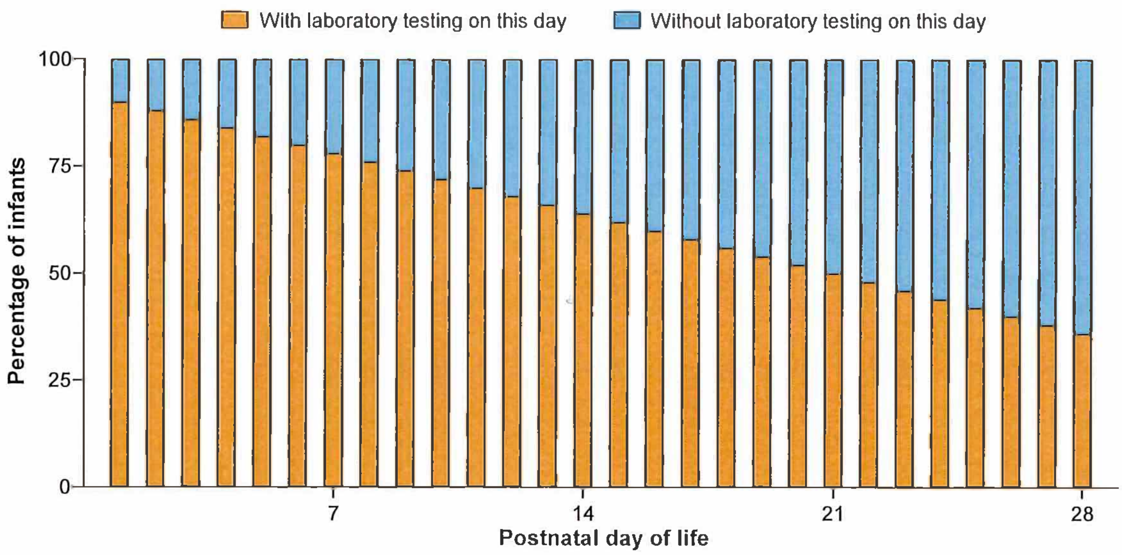

Example figure, stacked 100% bar.

5.2 Blood volume withdrawn per postnatal day

We will calculate the median (IQR) total blood volume withdrawn (in mL/kg birthweight) per postnatal day per infant. Infants will be included in the analysis for the postnatal days in which they were followed in study follow-up (from day 1 to 28, or part thereof).

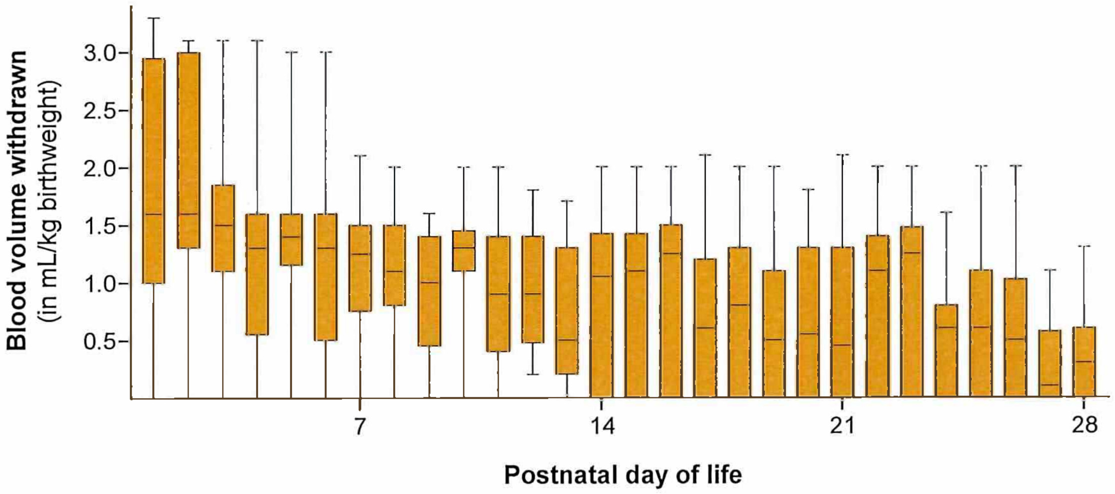

Example figure, boxplot median (IQR, min-max).

5.3 Cumulative blood volume withdrawn from postnatal day 1 to 28

The cumulative blood volume withdrawn from day 1 to day 28 will be calculated by summing the median bloods volume withdrawn per postnatal day per gestational age group. The corresponding IQRs will be calculated in an similar matter by summing the 25th and 75th percentiles, respectively.

We aim to present graphs starting from 0% from the total circulating blood volume, seperately per gestational age group. The total circulating volume for each GA stratum will be calculated by multiplying the median birth weight per gestation age group by 70 mL/kg. (2)

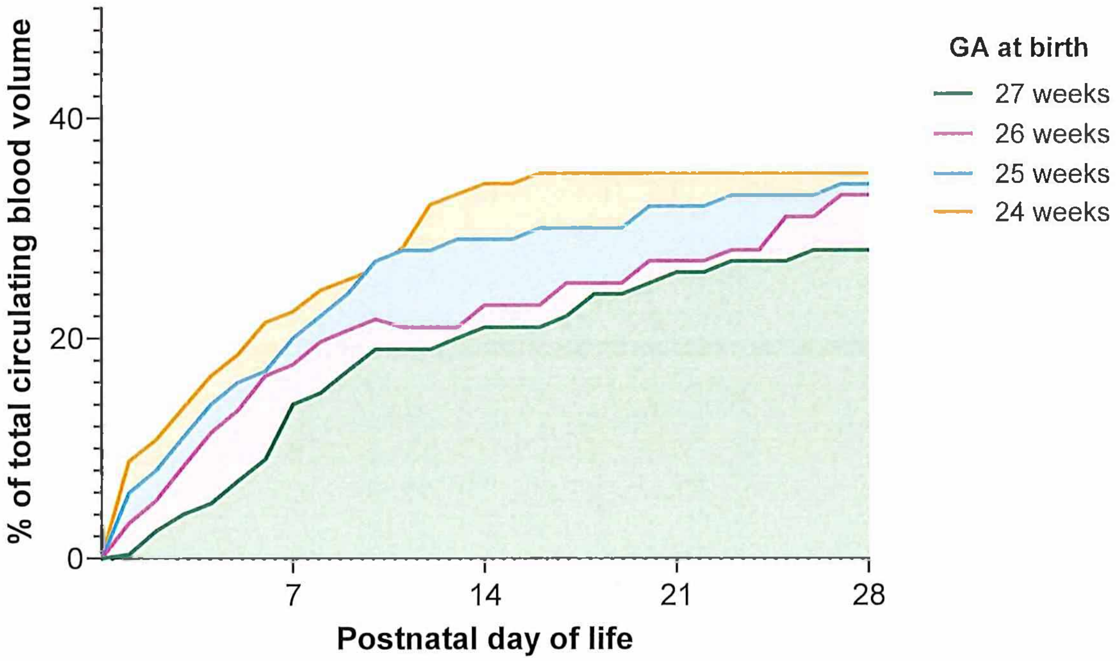

Example figure, plot starting at 0% of total circulating blood volume.

5.4 Number of laboratory tests and blood volume withdrawn on day 1 and 2

We will calculate the median number of laboratory tests conducted per infant and the total blood volume withdrawn for these tests per infant per country on postnatal days 1 and 2. These data will be presented in two ways, per infants using a scatter plot and per country by presenting the median total number of tests and the median volume withdrawn (see example figure).

This analysis aims to highlight the coherence between the volume of blood required per center and the number of laboratory tests performed. This approach will allow us to distinguish countries that perform more laboratory tests but require less blood for this, and vice versa. For this analysis, we will include data on postnatal days 1 and 2 for infants who were followed for at least these days. Although ideally we would have limited our analysis to the first 24 hours, we did not collect data on the time of birth. By including days 1 and 2, we ensure that we capture at least the first 24 hours for each infant.

We chose this timeframe for the following reasons: clinical differences are relatively limited during these first two days compared to later days as all infants mainly present with extremely prematurity. Additionally, most of the blood volume is withdrawn during the first two days of life, as reported by Counsilman et al (2020).

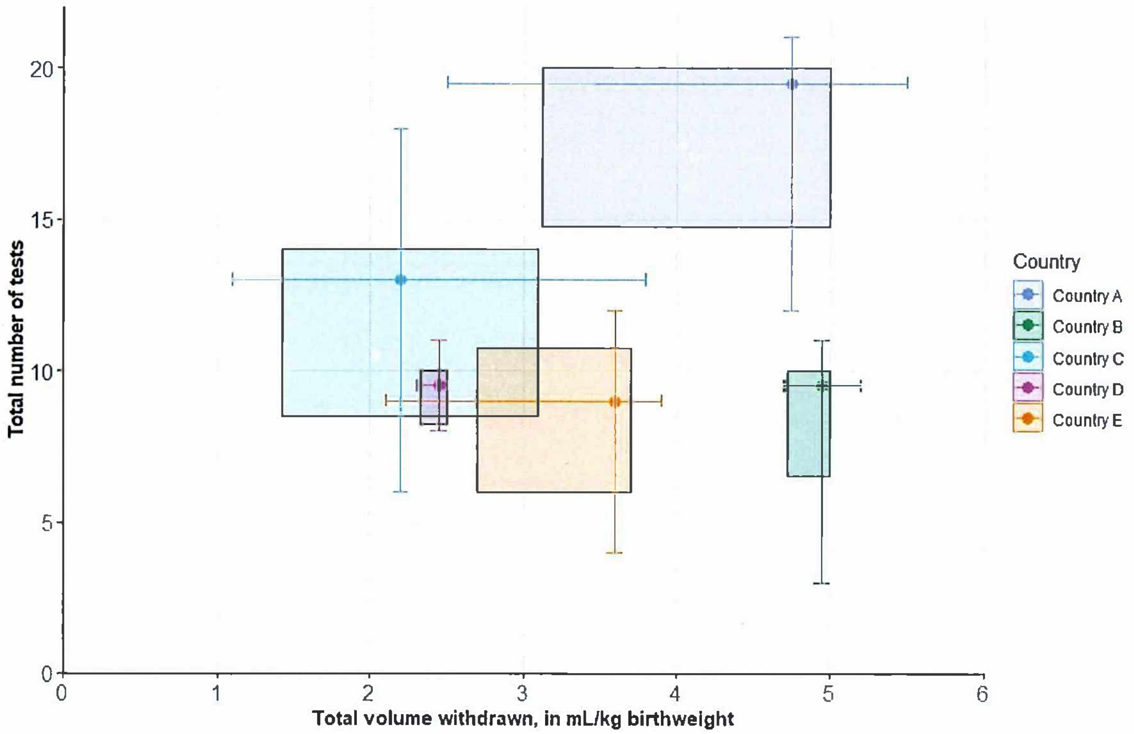

Example figure, double box plot with median (IQR).

5.5 Blood volume required per type of laboratory test

We will present the blood volume required per center per type of laboratory test, to highlight variation across Europe.

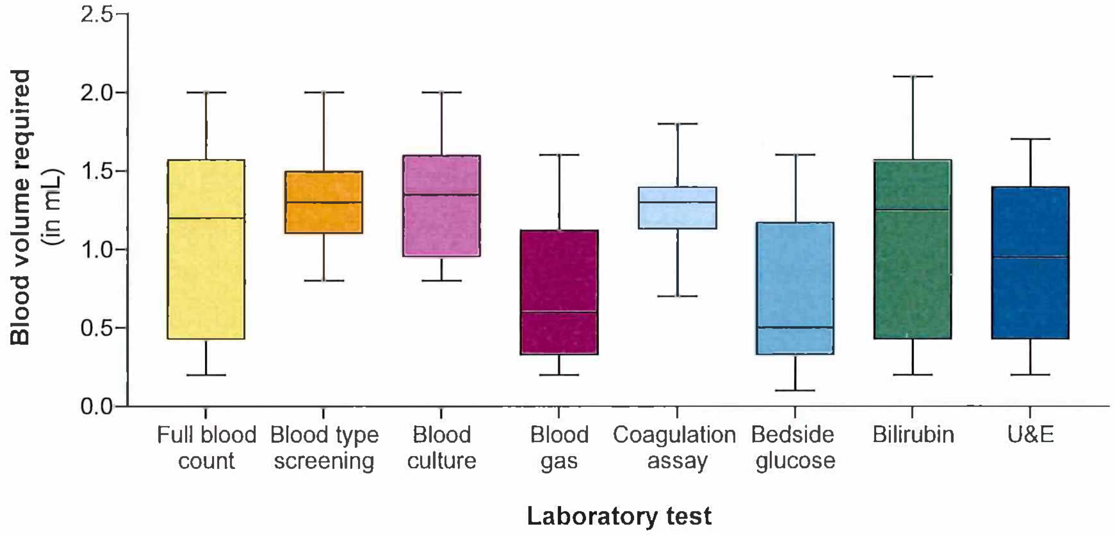

Example figure, boxplot median (IQR, min-max).

5.6 Total blood volume required for all laboratory tests combined

Under Section 5.5, we aim to highlight the variation among centers in the required blood volume per analyzer. However, that figure does not allow for assessment whether certain centers consistently require more or less blood across multiple analyzers, which would be insightful.

Therefore, for this outcome, we aim to calculate the total blood volume needed per center if all tests are conducted for a hypothetical work-up (consisting of 1x full blood count, 1x blood type screening, 1x blood culture, 1x blood gas, 1x coagulation assay (APTT/PT, INR or thromboelastography), 1x bedside glucose, 1x bilirubin, 1x urea and electrolytes (U&E)). The total volume needed per center will be presented stratified by country.

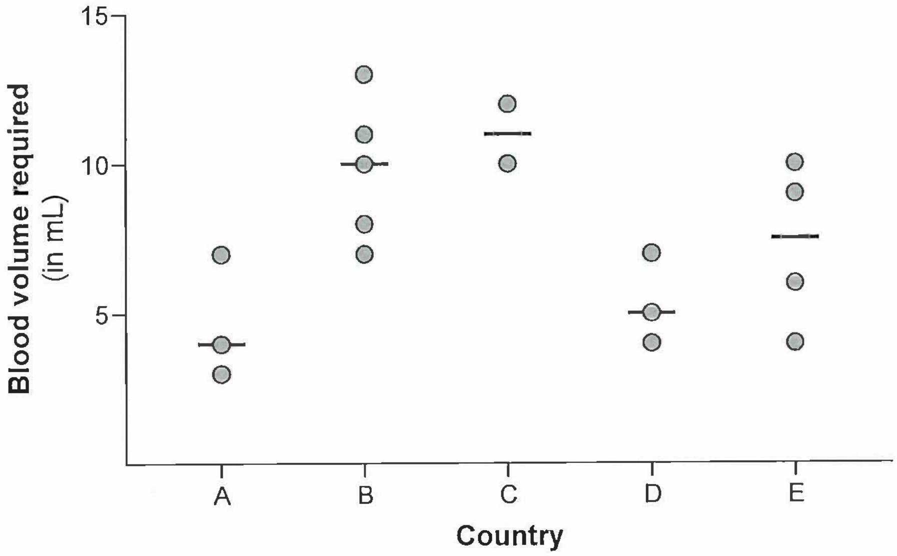

Example figure, required blood volume stratified per country.

6. STATEMENT

We hereby declare that this statistical analysis plan has been drafted prior to analysis of the INSPIRE data for this manuscript, and that this plan will be followed during the analysis phase. If any deviations from the plan are made, they will be incorporated and explained in an amendment.

Date:

Version:

Principal investigator:
Prof. dr. E. Lopriore

Clinical epidemiologist:
Prof. dr. Johanna van der Bom

Coordinating investigator:
Nina Houben

7. REFERENCES

1. Counsilman CE, Heeger LE, Tan R, Bekker V, Zwaginga JJ, Te Pas AB, et al. Iatrogenic blood loss in extreme preterm infants due to frequent laboratory tests and procedures. The journal of maternal-fetal & neonatal medicine : the official journal of the European Association of Perinatal Medicine, the Federation of Asia and Oceania Perinatal Societies, the International Society of Perinatal Obstet. 2019:1-6.
2. Lopriore E. The total volume of blood in an extremely preterm neonate is about the size of a double espresso. Acta Paediatr. 2023;112(12):2458-9.
